# Hypoxia Signaling and Circadian Disruption in and by Pheochromocytoma

**DOI:** 10.3389/fendo.2018.00612

**Published:** 2018-10-16

**Authors:** Mouna Tabebi, Peter Söderkvist, Lasse D. Jensen

**Affiliations:** ^1^Department of Clinical and Experimental Medicine, Linköping University, Linköping, Sweden; ^2^Department of Medicine and Health Science, Linköping University, Linköping, Sweden

**Keywords:** pheochromocytoma, circadian, hypoxia, cancer, blood pressure, catecholamines

## Abstract

Disruption of the daily (i.e., circadian) rhythms of cell metabolism, proliferation and blood perfusion is a hallmark of many cancer types, perhaps most clearly exemplified by the rare but detrimental pheochromocytomas. These tumors arise from genetic disruption of genes critical for hypoxia signaling, such as von Hippel-Lindau and hypoxia-inducible factor-2 or cellular metabolism, such as succinate dehydrogenase, which in turn impacts on the cellular circadian clock function by interfering with the Bmal1 and/or Clock transcription factors. While pheochromocytomas are often non-malignant, the resulting changes in cellular physiology are coupled to de-regulated production of catecholamines, which in turn disrupt circadian blood pressure variation and therefore circadian entrainment of other tissues. In this review we thoroughly discuss the molecular and physiological interplay between hypoxia signaling and the circadian clock in pheochromocytoma, and how this underlies endocrine disruption leading to loss of circadian blood pressure variation in the affected patients. We furthermore discuss potential avenues for targeting these tumor-specific pathophysiological mechanisms therapeutically in the future.

## Introduction

Cancer is a growing health threat exhibiting relentlessly increasing incidence due to changes in the pro-tumorigenic environmental stressors of our modern lifestyle as well as improved longevity, screening programs and diagnostic capabilities. This increase in incidence is particularly evident for rare, endocrine-disrupting cancers such as pheochromocytomas (PCCs), exhibiting an incidence of approximately 5 cases per million life-years in Scandinavia (1). As the pathology and treatment of PCC, as well as other rare cancers are poorly understood, there is a need for more investigations into these types of cancer. PCC is characterized by endocrine disruption in the patient, primarily evident as pathogenic, non-circadian blood pressure dysregulation (2). As such, PCC clearly interferes with circadian, endocrine regulation of cardiovascular physiology. On the other hand, disruptions in circadian physiology may also lead to stress and de-regulation of the chromaffin cells, potentially increasing the risk of PCC development (3). In addition, PCC pathology is often genetically linked to disrupted oxygen sensing and hypoxia signaling (1, 4), which in turn is intimately coupled to regulation of the cellular circadian clock (5) and to endocrine as well as local regulation of cardiovascular function and metabolism (6). As such, PCC represents a malignancy in which multiple aspects of hypoxia and circadian regulation of endocrine functions are affected, providing a strong case for understanding such interplay in more detail. In this review we discuss the molecular and physiological basis for crosstalk between the endocrine, metabolic and cardiovascular systems, hypoxia signaling and the circadian clock in the context of PCC, highlighting bilateral interactions between these systems, which in many cases are also relevant for other cancer types.

## Pheochromocytoma

Pheochromocytoma (PCC) is a catecholamine-producing tumor of chromaffin cells typically located in the adrenals. Most patients present with benign tumors, but as both benign and malignant tumors produce large amounts of endocrine-disrupting hormones, we will not differentiate between these two different classes in this review. They are characterized by an abnormally high production of catecholamines causing persistent or paroxysmal hypertension recurrent episodes of headache, palpitations and sweating, and an increased risk of cardiovascular disease (7). Germline and somatic genetic alterations in a growing number of genes are associated with PCC development and the major susceptibility genes are; the von Hippel–Lindau (*VHL*) tumor-suppressor protein, the rearranged during transfection (*RET*) protooncogene, the neurofibromatosis type 1 (NF1) tumor suppressor gene, genes encoding the four subunits (A, B, C, D) of the succinate dehydrogenase (*SDH*) complex and the gene encoding the enzyme responsible for flavination of the SDHA subunit (*SDHAF2*), and in rare occasions the egl-9 family hypoxia-inducible factor1/ Prolyl hydroxylase domain 2 protein (*EGLN1/PHD2*) tumor suppressor. Recently, three other genes, transmembrane protein 127 (*TMEM127*), MYC-associated factor X (*MAX*), and hypoxia-inducible factor 2α (*HIF2*α*/ EPAS1*) have been described to be mutated or amplified (1, 4, 8–10). The known germline and somatic mutations account for the pathogenesis of approximately 60% of PCC (7). Approximately, 30% of pheochromocytomas are hereditary and caused by mutations in well-known susceptibility genes (1, 11, 12). Interestingly, many sporadic pheochromocytomas have somatic mutations limited to only one of these hereditary genes, indicating that they may be the main driver. However, the cause of many sporadic tumors is still unknown (1, 8, 13). The most commonly mutated genes are found in the SDH complex, *SDHD* (87.1%), followed by *SDHAF2* (6.7%), *SDHB* (5.9%) and *SDHC* (0.3%). About 20% of tumors have mutations in the *NF1* gene, about 14% in the *RET* and *VHL* genes, 5-10% in *HRAS* but only 2.5 and 1.65% of the tumors present *HIF2A* and *MAX* mutations, respectively(7, 14–16).

**Figure 1:**
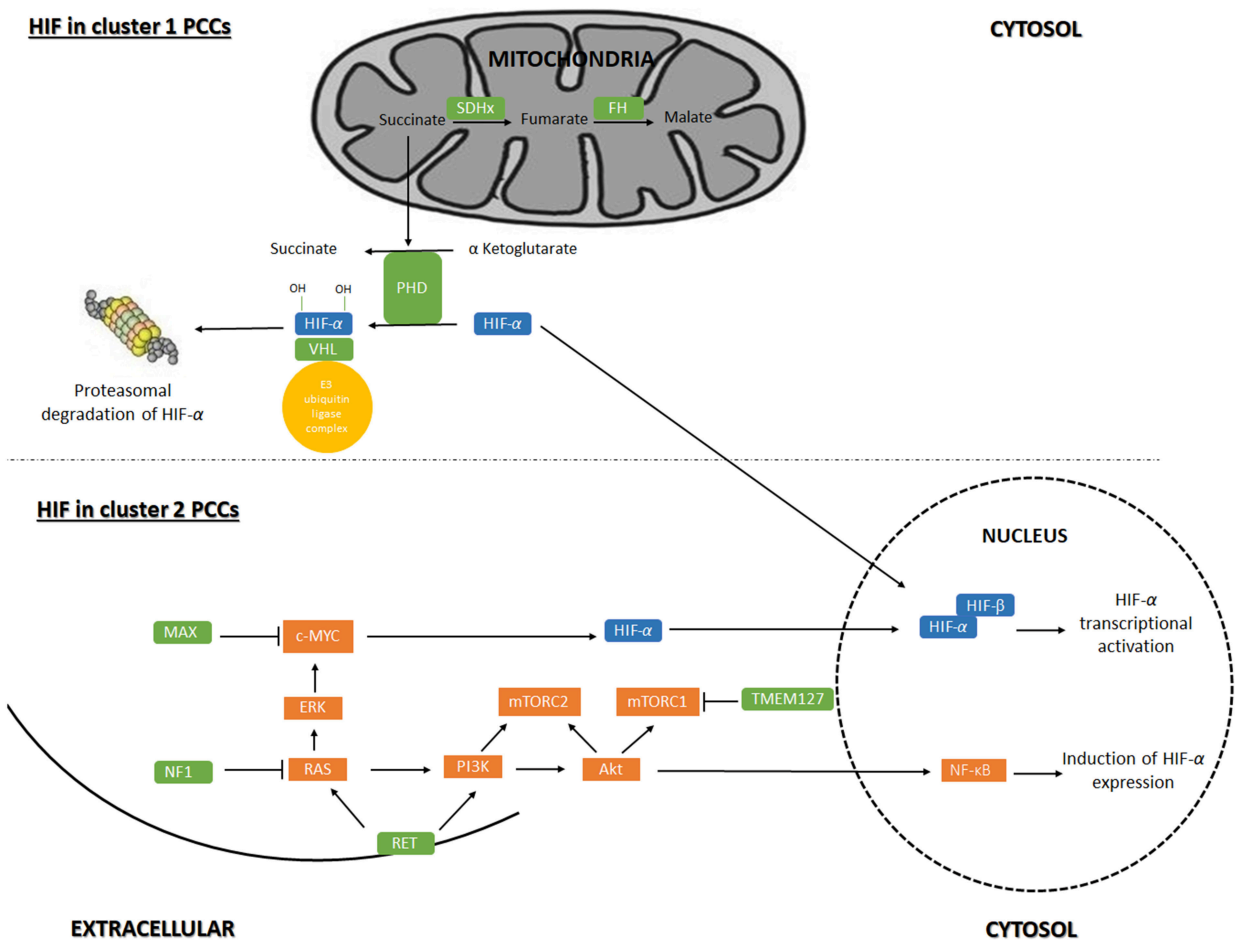
Regulation of gene-expression by Hypoxia-inducible factor signaling in pheochromocytoma. Hypoxia-inducible factor (HIF) signaling in cluster 1 and cluster 2 pheochromocytoma (PCC). Akt, RAC-alpha serine/threonine-protein kinase; c-Myc, Myc proto oncogene; ERK, extracellular signal-regulated kinase; MAX, myc-associated factor X; mTORC1, mammalian target of rapamycin complex 1; mTORC2, mammalian target of rapamycin complex 2; NF1, neurofibromin 1; NF-κB, nuclear factor kappa B; PHD, prolyl hydroxylase domain protein; PI3K, phosphoinositide 3-kinase; VHL, von Hippel-Lindau; Ras = rat sarcoma oncogene; RET, Ret proto-oncogene; SDHx, succinate dehydrogenase; TMEM127, transmembrane protein 127.

Based on the main signaling pathway signatures resulting from hereditary and sporadic pheochromocytoma gene mutations, pheochromocytomas are divided in two main gene expression clusters (17–19): The first group, pseudo-hypoxia cluster, included tumors carrying *SDHx* (cluster 1A) and *VHL* (cluster 1B) mutations, which accounts for 30% of the sporadic tumors. In addition, mutations in fumarate hydratase (*FH)* (cluster 1A) and *HIF2A* (cluster 1B) are characterized by transcription signatures suggesting reduced oxidoreductase activity and increased hypoxia and angiogenesis (Figure 1). The second cluster represents expression signatures of mutated genes in kinase receptor signaling and protein translocation pathways, i.e., the *RET-* and *NF1*- related pheochromocytomas and includes 70% of the sporadic tumors. Cluster 2 can be divided into subcluster 2A, 2B, 2C and 2D. Subcluster 2A comprises *RET, MAX, NF1* and *TMEM127* mutated tumors, subcluster 2B and 2C include sporadic tumors and cluster 2D include tumors lacking known mutations related to PCC. Cluster 2 mediate translation initiation, protein synthesis, adrenergic metabolism, neural/neuroendocrine differentiation and kinase signaling (Figure 1).

### HIF in cluster 1 PCCs

Cluster 1 tumors are mostly extra-adrenal, expect for *VHL* tumors which usually are adrenal (20). SDH-related tumors, especially those associated with *SDHB*, are more aggressive and prone to metastasis than VHL PCCs that shows a low risk of malignancy (21). Mutations in *VHL* cause VHL disease. In presence of oxygen, The VHL protein (pVHL) is the substrate recognition unit of the VBC E3 ubiquitin ligase complex that target HIF for proteasomal degradation. HIF-1α and−2α are hydroxylated by PHD under normoxic conditions, which is recognized by pVHL for ubiquitination (22). Mutations in *VHL* that cause VHL disease leads to the production of a pVHL that is not able to recognize hydroxylated HIFs resulting in stabilized and therefore increased levels of HIF and HIF-associated transcription of target genes (18, 23–25).

SDH-related tumorigenesis is also associated with the HIF pathway (activation and stability) (26). *SDHx* is a part of complex 2 in the mitochondrial respiratory chain and when mutated disrupts the activity and stability of the SDH enzyme resulting in succinate accumulation. As PHD hydroxylation activity requires the conversion of α-ketoglutarate to succinate (27), succinate accumulation inhibits PHD-catalyzed HIF-1/2α hydroxylation (28), potentially leading to hypoxia signaling in the PCC cells.

Several findings support the leading role of HIF-2α in development and progression of cluster 1 tumors (18, 29). Patients with PCCs carrying *SDHB* or *SDHD* mutations, present an overexpression of HIF-2α and transcriptional targets like VEGF found in PCCs (29, 30). In addition, the accumulation of succinate as a result of tumors carrying *FH* mutations displays the CpG island methylator phenotype (CIMP), the same pattern of epigenetic deregulation as SDHB-mutated malignant PCC (31).

All PCC with *HIF2A/EPAS1* mutations showed higher expression of EPAS1 and HIF-2α target genes e.g *VEGF* (4, 10, 15, 32). However, the reason or the mechanisms are still not clear. The mutational analysis of *EPAS1* gene by sequencing of exon 9 and 12, which contain the two hydroxylation sites involved in HIF-2α degradation, showed that more than 12% of PCC with isolated, non-familial tumors present several missense *EPAS1* mutations in this region (4, 33).

### HIF in cluster 2 PCCs

Cluster 2 tumors are mostly adrenal, except for MAX/related tumors, with a low risk of malignancy (14). In MEN2 and NF1 syndromic cases, the RET and NF1inactivating mutations causes the activation of Ras/MAPK and PI3K/AKT pathways (34). In tumors caused by *TMEM127* and *MAX* mutations, mTORC activation could increase HIF-1/2α levels (35): Mutations in *TMEM127* gene lead to enhancement of mTORC signaling in a Ras- and PI3K/AKT-independent manner(36). Mutations in *MAX* deregulate c-MYC signaling of which HIF-1α is a downstream target (33). Thus, crosstalk between the Myc/MAX/MXD1 (MAX-interacting protein 1) and Ras/PIK3/AKT1/mTORC has been proposed (37), suggesting that MAX affects the mTORC pathway as well (18). *K-RAS* and *H-RAS* mutations were found in PCCs, causing the activation of the Ras/RAF/ERK signaling pathway, a part of the Ras/MAPK pathway (38). Ras/MAPK pathway activation leads to an increase in HIF-1/2α signaling and the transcription of HIF target genes.

Despite the characterization of these two main groups a comprehensive molecular distinction between VHL- and SDHx- related tumors on one hand and RET- and NF1- related tumors on the other hand, has not been achieved, as pseudohypoxia signaling may also be present in the latter sub-group. However, VHL tumors could be distinguished, for example, from SDHx tumors by transcription profiling, using restricted lists of genes involved in hypoxia (19) or glycolytic (23) pathways.

## HIF in PCC development

Hypoxia-inducible factors belong to the basic helix loop helix-PER-ARNT-SIM (bHLH-PAS) family of transcriptional regulators and have a similar protein domain structure as circadian rhythm regulators (39). HIFs are highly conserved proteins composed of α and β subunits, that respond to changes in tissue oxygen concentration. The HIF-α subunits (HIF-1α, HIF-2α and HIF-3α) are stabilized by hypoxia, as described above, whereas the HIF-β subunit is constitutively expressed (39, 40). Under hypoxia, stabilized HIF-α subunits dimerize with ARNT (aryl hydrocarbon nuclear translocator) and activate transcription of genes involved in the hypoxia response such as apoptosis, glycolysis, cell growth and proliferation (41).

The HIF pathway was previously found to be dysfunctional in PCC tumors (26). Indeed, most of the hereditary PCCs are related to the hypoxia signaling pathway by increasing the stability of HIF, through the mutations within the *FH, SDHx, SDHAF2, VHL*, and *PHD2* genes. *SDHx* mutations increase the stability of HIFs and the expression of their targets (15, 30). The *SDHx* gene mutations result in the functional inactivation of the SDH complex results in the intracellular accumulation of succinate, which is converted by the PHDs from α-ketoglutarate. This accumulated substrate in turn inhibits the PHDs because of its structural similarity with α-ketoglutarate. Since the stability of the HIF-α subunits depends on their hydroxylation by PHDs, the *SDHx* mutation leading to inhibition of PHD will prevent degradation of HIF(42). In addition, the knockdown of *SDHA* or *SDHB* in cell lines leads to the inhibition of α-ketoglutarate-dependent enzymes. This leads to hypermethylation of histones and DNA which also result in HIF-α accumulation and strong expression of HIF targets (42).

Several recent studies have also proposed that the PCCs mutations of *NF1, RET, HRAS* resulting in a cluster 2 type of gene expression pattern also connects to HIF signaling and acts on the same signaling network, resulting in i.e decreased apoptosis during chromaffin cell development and tumor formation. Moreover, cluster 2 gene mutations seem to enhance HIF- 2α signaling in cells (18, 43, 44).

Deregulation of HIF-α, specifically HIF-2α, has long been implicated in tumorigenesis and in poor prognosis of other human cancers as well as PCC (25). In particular, HIF-2α activation was shown to be both necessary and sufficient for development of VHL-null renal cell carcinomas (45, 46).

## Hypoxia and the circadian clock–to sides of the same coin?

Hypoxia signaling and the circadian clock are closely related and heavily cross-communicating pathways (5). Both pathways are evolutionarily ancient, presenting the first molecular mechanisms allowing living organisms to adapt to environmental stimuli on earth (light/dark cycles and the strongly oxidative environment respectively) (47–50). Interestingly, the core components of these pathways belong to the same family of transcription factors and are regulated in a very similar fashion. Both the central mediators of hypoxia signaling, HIF-1α, HIF-2α, HIF-1β (ARNT), discussed above, and the core clock proteins Clock, Bmal1 and Period belong to the bHLH-PAS family of heterodimerizing transcription factors. They furthermore interact with each other to elicit induction or repression of transcription at the promotor regions of target genes (5, 51). This implies that HIF-1β can interact with either HIF-1α or Clock, and Bmal1 can interact with either Clock or HIF-1β leading to the formation of either canonical hypoxia or circadian transcription factors, or mixed hypoxia/circadian transcription factors respectively(51, 52). In addition, the HIF-consensus DNA-binding motif ((A/G)CGTG), called the hypoxia-responsive element (HRE) (40) is similar to and included within the clock-consensus DNA-binding motif CACGTG, called the E-box (53). E-boxes are therefore also HREs, meaning that HIF-1 compete with Bmal1/Clock heterodimers for binding and activation of clock-controlled gene transcription (6, 54) and some HREs are also E-boxes, meaning that Bmal1/Clock heterodimers may induce or disrupt HIF-mediated transcription (55). Accordingly, hypoxia-induced signaling is disrupted in circadian clock-deficient animals and circadian signaling is disrupted by hypoxia (56). As an example of this cross-talk, one of the archetypical hypoxia-induced genes coding for vascular endothelial growth factor (VEGF) was recently found to be completely subject to circadian control by Bmal1 and negatively regulated by Period2 in zebrafish embryos, leading to a circadian regulation of developmental angiogenesis(57, 58). Furthermore, the DNA-binding affinities of heterodimeric circadian transcription factor complexes Clock1:Bmal1 and Clock2:Bmal1 are highly sensitive to redox-modifications, exhibiting much higher E-box binding and transcriptional activity in the reduced state, suggesting that cellular oxidative stress, which is often associated with prolonged hypoxia, impairs the circadian clock (59). The disruption of circadian signaling by hypoxia was recently found to depend on lactic-acid mediated acidification, via disruption of mTORC1 signaling, possibly pin-pointing the molecular intersection between these two pathways and suggesting that their intimate cross-talk can be uncoupled by interfering with lactic acid metabolism and mTOR/mTORC1 activity (60). Additional similarities such as the importance of ubiquitin-mediated destruction of circadian factors (61) and their cross-talk through transcription-translation negative feedback loops (5) have been excellently reviewed elsewhere.

## Pheochromocytomas affect circadian rhythms

Epidemiological evidence have provided correlative evidence linking sleep disturbances in night shift workers to increased incidence and poor prognosis in hormonal cancers (62–64). The circadian rhythm is tightly connected to cell division and act as an additional checkpoint to determine the timing of mitosis. Certain stem cells may also synchronize their cell division due to rhythmic secretion of mitogens from adjacent cells. The circadian clock genes thus act as tumor suppressor genes and disruption of expression uncouples the control of the cell cycle from the circadian clock and promotes carcinogenesis (65). The suppression of circadian clock transcription factors in breast and prostate cancer cells is important for their malignant transformation and deletion of PER1 and PER2 in mice results in salivary gland tumor formation (62). Several oncogenic pathways such as cell cycle regulators and tumor suppressors are deregulated and associated to the circadian clock (66), such as the increased activity of Ras pathway (67). Overexpression of MYC is directly controlled by circadian proteins and upregulated in *Per2* mutant mice and was recently shown to be a co-repressor of EMT (Epithelial-Mesenchymal Transition) (68). Further, upregulated *MYC* in different cancers silence the circadian clock and promotes cell proliferation while downregulation of MYC activates the clock and reduces proliferation (69).

As mentioned above, PCC is a pseudohypoxic tumor due to genetic disruption of the hypoxia signaling pathway (i.e., mutation in VHL), or due to mutations causing deficiencies in metabolic intermediates required for hydroxylation-mediated degradation of HIF1α. However, PCC is also a tumor type with high density, heavily disrupted and pathological blood vessels regardless of malignancy (70), indicating that blood flow is disrupted, further contributing to the hypoxic and acidic status of the tissue. This likely disrupts the circadian entrainment of the PCC cells (and non-malignant PCC stroma cells) by endocrine factors, contributing to disease progression.

The process of malignant transformation can furthermore affect circadian rhythms in distal, non-malignant tissues and organs, by introducing an imbalance in metabolic homeostasis of the body (65). For example, arrhythmic liver metastases of colorectal cancer were found to shift the phase of clock gene expression in healthy liver tissue (6). Similarly, a study on mice with pulmonary adenocarcinomas demonstrated that these caused alterations in hepatic circadian metabolism. In fact, the expression of the basic genes of the clock remains unchanged, while lung tumors specifically affected the oscillations of genes and molecules related to liver metabolism (71).

Circadian rhythms in the body are also affected by tumors that produce hormones such as PCC. Patients with adrenaline-producing PCC have circadian changes in blood pressure (BP) (2, 72). The attenuation of circadian blood pressure variation was more pronounced in PCC compared, for example, to steroid-induced adrenal hypertension (primary aldosteronism and Cushing syndrome). In fact, elevated catecholamine production in primary aldosteronism likely maintains a circadian rhythm, which is not the case in PCC, and this could be the reason for why diminished diurnal BP variation can only be observed in PCC, in spite of very high catecholamine production in both types of patients (72–74). Circadian rhythms in catecholamine production and secretion are closely linked to diurnal blood pressure variation in healthy adults (75, 76). Moreover, it was previously reported that normal subjects show a circadian rhythm in BP, with the highest values in the morning and the lowest at night (46). Asymptomatic subjects with pheochromocytoma and controls group did not differ in the office BP, but they differed significantly in the 24-h systolic and diastolic BP and also in the relative systolic and diastolic night-time BP decline (2).

In the case of PCC, alterations of sympathetic vascular regulation is the main cause of the pathogenesis of orthostatic hypotension, whereas cardiac vagal regulation acts to compensate for it (77). In fact, the tendency to the orthostatic hypotension with normal baroreflex is the most important factor contributing to the change in altered or inverted circadian BP. It causes a decrease in BP and increases the heart rate during the active period of the day more significantly than during the night (72, 77, 78). These observations reflect higher sympathetic tonus than in normosensitive and hypertensive patients.

Zelinka et al. showed that circadian BP variation is reduced in both patients with asymptomatic normotensive PCC and patients with typical, hypertensive PCC to the same degree (2). However, only the mechanisms responsible for circadian BP disruption in hypertensive patients with PCC has been described previously (2, 74), and the mechanisms by which PCC leads to loss of circadian BP therefore remains incompletely understood (74). The reduction of circadian BP rhythm may be due to the partial desensitization of the cardiovascular system to catecholamines in PCC is insufficient (2, 72, 79).

Depending on expression of biosynthetic enzymes (80, 81), constitutive or regulated secretory pathways (82), PCC tissues produce different concentrations of catecholamines. Recently, new evidences confirmed the existence of mutation-dependent biochemical phenotypes in PCCs: all MEN2 and NF1 tumors are characterized by increased plasma concentrations of metanephrine. Usually, VHL tumors present with increases in normetanephrine and 70% of patients with *SDH* mutations are characterized by additional increases in methoxytyramine (82). However, some patients with high levels of circulating catecholamines associated with nocturnal changes in blood pressure, are not hypertensive, suggesting that other regulatory mechanisms associated with circadian fluctuation of blood pressure, such as the sympathetic nervous system and/or the hypothalamic-pituitary-adrenal system are still active and important in PCC patients (83). It should also be noted that about 15–20% of patients with PCC, presented a normal concentration of catecholamines (79). Most of these cases are explained by the non-secretion of the significant amounts of catecholamines which are called “non-functional” or “silent” PCC (81). The lack of catecholamine secretion in biochemically silent PCC is not due to defects in the mechanisms of storage or secretion of catecholamines but to the absence of tyrosine hydroxylase (TH), the initial and rate limiting enzyme in catecholamine formation. This phenotype appears to be associated with mutations of the SDHB gene (84). In most PCC cases, however, previously modified circadian BP variation is improved after successful tumor removal, or medical treatment (72).

## Conclusion and future prospects

Pheochromocytomas are chromaffin cell tumors that arise from the adrenal medulla. The dysfunction of genes involved in the cellular response to hypoxia, such as VHL, EGL nine homolog 1, and the succinate dehydrogenase (SDH) genes, leads to direct abrogation of hypoxia-inducible factor (HIF) degradation, resulting in a pseudo-hypoxia state implicated in PCC development (33). HIFα signaling is hypothesized to be an effective signaling pathway involved in the pathogenesis of hereditary and sporadic PCC and other neuroendocrine tumors. HIFα is a very promising therapeutic target, particularly for aggressive and metastatic tumors where the introduction of HIF-2α inhibitors is of prime interest at present. Recently, many agents affecting HIF1α signaling have been introduced, leading to different results depending on a cancer HIFα phenotype (85). Otherwise, drugs targeting the HIF-2α signaling pathways have not yet been described but are currently under development (86, 87).

## Author contributions

PS and LJ outlined the content and supervised the Project. MT and LJ performed research and wrote the manuscript. All authors read, revised and approved the manuscript.

### Conflict of interest statement

The authors declare that the research was conducted in the absence of any commercial or financial relationships that could be construed as a potential conflict of interest.
